# An Empirical Study of Applying Statistical Disclosure Control Methods to Public Health Research

**DOI:** 10.3390/ijerph16224519

**Published:** 2019-11-15

**Authors:** Amanda M. Y. Chu, Benson S. Y. Lam, Agnes Tiwari, Mike K. P. So

**Affiliations:** 1 Department of Social Sciences, The Education University of Hong Kong, Tai Po, Hong Kong, China; 2 Department of Mathematics and Statistics, The Hang Seng University of Hong Kong, Shatin, Hong Kong, China; 3 School of Nursing, The University of Hong Kong, Pokfulam Road, Hong Kong, China; 4 School of Nursing, Hong Kong Sanatorium & Hospital, Hong Kong, China; 5 Department of Information Systems, Business Statistics and Operations Management, The Hong Kong University of Science and Technology, Clear Water Bay, Hong Kong, China

**Keywords:** data perturbation, data privacy, data utility, health care, risk

## Abstract

Patient data or information collected from public health and health care surveys are of great research value. Usually, the data contain sensitive personal information. Doctors, nurses, or researchers in the public health and health care sector do not analyze the available datasets or survey data on their own, and may outsource the tasks to third parties. Even though all identifiers such as names and ID card numbers are removed, there may still be some occasions in which an individual can be re-identified via the demographic or particular information provided in the datasets. Such data privacy issues can become an obstacle in health-related research. Statistical disclosure control (SDC) is a useful technique used to resolve this problem by masking and designing released data based on the original data. Whilst ensuring the released data can satisfy the needs of researchers for data analysis, there is high protection of the original data from disclosure. In this research, we discuss the statistical properties of two SDC methods: the General Additive Data Perturbation (GADP) method and the Gaussian Copula General Additive Data Perturbation (CGADP) method. An empirical study is provided to demonstrate how we can apply these two SDC methods in public health research.

## 1. Introduction

Patient data or information collected from public health and health care surveys are of great value for safeguarding human physical and mental health, as well as improving medical services and relevant social policies [1]. For example, Hodge [2] suggests that the information could be used to improve efficiency within the health care system, drive public policy development and administration, and improve the conduct of medical research. It is understandable that the more detailed and accurate the data released to researchers for analysis are, the more research findings can be obtained. However, data related to public health and health care usually contain a lot of personal information. For example, the survey data in Mercuri [3] include identifiers such as physicians’ subjective assessments of personality, income, mental state, history of medical diagnosis, treatments, medication history, dietary habits, and sexual preference. If the full original health-related datasets are released to researchers for analysis without any changes, the privacy of patients or respondents may be compromised. Even though the identifiers have been removed, there may still have some occasions that an individual can be re-identified from the information in the datasets. Therefore, data providers have to pay high attention to balancing data utility and the data privacy.

Many scholars have discussed the methods used to control the disclosure of confidential information. One fundamental method is the restriction of data access and disclosure via signing a licensing agreement or limiting data access. Guttman and Stern [4] suggested that licensing agreements are too insecure and risky, and that limiting data access limits the research scope too. Abowd and Lane [5] used a three-tiered approach, including developing inference-valid synthetic microdata, maintaining data in secure and restricted access environments, and developing a research data center network to protect the confidentiality of the data. There is another basic method to protect the data privacy, and we denote it as data exclusion, a typical method of which has been proposed by Sweeney [6]. Using this method, we can ensure that at least *k* attributes included in any given original health-related data cannot be re-identified. In addition, Sweeney [7] has provided a computer program named Datafly to guarantee anonymity when sharing medical data, and Berman [8] has provided a general algorithm that removes all identifiers and private information. As for generating a synthetic microdata method, readers can read Drechsler [9], Alfons et al. [10], and Templ and Filzmoser [11] for more details.

Moving one step forward from the data exclusion method, there are a number of data transformation methods that belong to the class of statistical disclosure control (SDC) methods that can protect the privacy of the original data. Some examples of SDC method are microaggregation by Domingo-Ferrer and Mateo-Sanz [12], data perturbation by Muralidhar et al. [13], data swapping by Carlson and Salabasis [14], and data shuffling by Muralidhar and Sarathy [15]. Some scholars have attempted to apply data exclusion or SDC to control disclosure and protect privacy in medical and health care data. El Emam and Dankar [16] applied a *k*-anonymity anonymization approach to a medical dataset so that the released data was identifiable to at least *k* individuals. Park et al. [17] provided a data perturbation algorithm called a perturbed Gibbs sampler, and applied it to patient data.

In this research, we focus on data perturbation, which is a classic example of data transformation. Generally, data perturbation protects the privacy of data by adding proper noise to the original data. It makes irreversible modifications to the original data whilst preserving the statistical information we are interested in. Many different perturbation methods, from simple to advanced, have been developed. Traub et al. [18] proposed an Adding Independent Noise (AIN) method, which is the fundamental one. Kim [19] improved the AIN method and proposed the Adding Correlated Noise (ACN) method. Tendick and Matloff [20] improved the ACN method and developed the Adding Bias Corrected Correlated Noise (ABCCN) method. Muralidhar el at. [13] suggested the General Additive Data Perturbation (GADP) method and Sarathy et al. [21] developed a GADP method based on the copula function and proposed the Copula General Additive Data Perturbation (CGADP) method, which includes the Gaussian CGADP method. In this research, we focus on the GADP method and the Gaussian CGADP method, which can be applied in a wider range of data related to health care. Through an empirical study, we demonstrate how these two methods generated perturbed data for confidential attributes in a healthcare study to protect the privacy of the respondents, whilst retaining the statistical information of the original database. This is important as healthcare research always includes confidential data, and it allows the perturbed database to be used for further research and analysis without the need of access to the original database.

The remainder of this paper is organized as follows. Section 2 discusses the statistical properties of the GADP and Gaussian CGADP methods. Section 3 presents an empirical study to describe how to apply the GADP and Gaussian CGADP methods to public health research. Section 4 concludes.

## 2. Methodology

SDC method generally refers to a class of methods based on probability and statistical theories used to protect data privacy from being disclosed. In this section, we discuss the statistical properties of two perturbation methods, the GADP method and the Copula perturbation method.

### 2.1. General Additive Data Perturbation Method

The GADP method was first introduced by Muralidhar et al. [13]. The method provides data information including means and co-variance to researchers for further analysis whilst protecting privacy by perturbing sensitive information such as health care information of data with additive noise. However, this method assumes that the sensitive information follows a normal distribution, which limits its applicability to real-world problems. This is explained via the following medical survey data.

Generally, the original health-related data collected by doctors, nurses, and researchers often includes many attributes. For example, a set of medical survey data for patients in a hospital includes $X_{1}$ (carrying human immunodeficiency viruses (HIV)), $S_{1}$ (height), $S_{2}$ (weight), $S_{3}$ (age), $S_{4}$ (name), and $S_{5}$ (address). From a research perspective, this original dataset is valuable for studying factors related to HIV. Before releasing the original data to other researchers for analysis, the hospital or data owner often remove attributes like $S_{4}\;\mathrm{and}\;S_{5}$, which may easily cause privacy disclosure issues. However, removing only these variables is insufficient for protecting the privacy of data, and there is still a possibility of data privacy disclosure due to the information provided from other attributes. For example, if a very tall and fat man in a particular ward carries HIV, it is not difficult to re-identify this patient by combining information from other attributes. This causes concerns to data privacy. To address this concern, it is better for the hospitals or data owners to apply the GADP method to the original data before releasing it to third parties for analysis. The GADP method divides attributes into two categories: confidential attributes and non-confidential attributions. In the example above, variable $X_{1}$ (HIV) is treated as the confidential attribute, and $S_{1}$ (height), $S_{2}$ (weight), and $S_{3}$ (age) are treated as non-confidential attributes, which are denoted as $X=\left(X_{1}\right)$ and $S=\left(S_{1},S_{2},S_{3}\right),$ respectively. For non-confidential attributes in S, the GADP method releases them directly. However, for confidential attribute X, the GADP method generates perturbed data Y and releases Y instead of X to prevent privacy disclosure. In other words, when we use the GADP method, the finally released data are (Y,S). Furthermore, the data (Y,S) released by the GADP method has two important properties:

1. the mean and standard deviation of each attribute of (Y,S) is same as those of (X,S) in expectation; and
2. the correlation matrix of (Y,S) is same as that of (X,S) in expectation.

So the GADP method protects data privacy without misleading researchers to get wrong conclusions. Before discussing how to generate the perturbed data *Y* by applying GADP method, we introduce some necessary symbols and assumptions.

#### 2.1.1. Symbols

1. $\mu_{X},\mu_{S},\mu_{Y}$ are the mean vectors of X,S,Y, respectively; and
2. $\Sigma_{XX},\Sigma_{SS},\Sigma_{YY}$ are the variance-covariance matrices of X,S,Y, respectively, while $\Sigma_{XY}$ is the variance-covariance matrices between X and Y—which is similar to $\Sigma_{YX},\Sigma_{XS},\Sigma_{SX},\Sigma_{SY},\Sigma_{YS}$—and $\Sigma_{XY}=\Sigma_{YX}^{\prime },\Sigma_{XS}=\Sigma_{SX}^{\prime },\Sigma_{SY}=\Sigma_{YS}^{\prime }$.

#### 2.1.2. Assumptions

1. $\mu_{X}=\mu_{Y},\Sigma_{YY}=\Sigma_{XX}$, and $\Sigma_{YS}=\Sigma_{XS}$; and
2. all marginal distributions of each attribute and the joint distribution of all attributes are normal distribution.

Under these assumptions, if we take Y,X,S as a random vector (Y,X,S), its joint distribution will be as follows:

(1) $$\left(\begin{array}{c} Y\\ X\\ S \end{array}\right)∼N\left(\left(\begin{array}{c} \mu_{Y}\\ \mu_{X}\\ \mu_{S} \end{array}\right),\left(\begin{array}{ccc} \Sigma_{YY} & \Sigma_{YX} & \Sigma_{YS}\\ \Sigma_{XY} & \Sigma_{XX} & \Sigma_{XS}\\ \Sigma_{SY} & \Sigma_{SX} & \Sigma_{SS} \end{array}\right)\right)$$

Furthermore, it is easy to know that the joint distribution of Y and S is same with the joint distribution of X and S. Based on the conditional probability, the GADP method generates the perturbed data $Y_{i},i=1,\ldots ,N$ for each given $U_{i}=\left(X_{i},S_{i}\right),i=1,\ldots ,N$. In other words, $Y_{i},i=1,\ldots ,N$ is the N samples drawn from the following conditional distribution:

(2) $$Y|U=U_{i}∼N\left(\mu_{X}+\Sigma_{YU}\Sigma_{UU}^{-1}\left(U_{i}-\mu_{U}\right),\Sigma_{XX}-\Sigma_{YU}\Sigma_{UU}^{-1}\Sigma_{UY}\right),$$

where

(3) $$\mu_{U}=\left(\mu_{X},\mu_{S}\right);\Sigma_{YU}=\left(\Sigma_{YX},\Sigma_{YS}\right);\Sigma_{UY}=\Sigma_{YU}^{\prime };\Sigma_{UU}=\left(\begin{array}{cc} \Sigma_{XX} & \Sigma_{XS}\\ \Sigma_{SX} & \Sigma_{SS} \end{array}\right).$$

**Figure 1:**
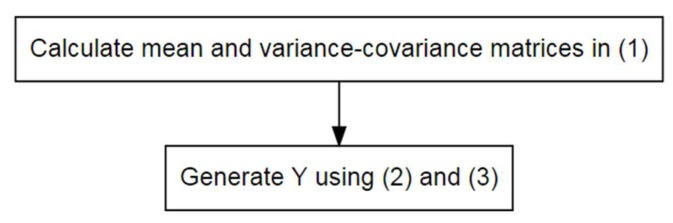
Procedure in applying GADP.

Finally, only the perturbed data Y and non-confidential data S are released to researchers, so the GADP method effectively protects the privacy of the original data whilst ensuring that the correlation of (X,S) remains unchanged. However, the GADP method has a shortcoming, that is, the assumption of normal distribution. In practice, the original data are generally very complex and cannot meet the assumption of normal distribution. To relax the assumption of the GADP method, Sarathy et al. [17] proposed a perturbation method based on a copula function. Figure 1 summarizes the procedure in applying GADP. 

### 2.2. Copula General Additive Data Perturbation Method

As mentioned above, the GADP method proposed by Muralidhar el at. [13] performs well when the underlying distribution of the data is jointly normal, but this assumption is too strong to put into practice. When the assumptions are not met, the results obtained based on the released data from the GADP method are distorted. A typical example is a set of bank survey data studied by Muralidhar et al. [13]. The bank survey data includes five attributes $X_{1}$ (home equity), $X_{2}$ (stock), $X_{3}$ (liabilities), $S_{1}$ (saving), and $S_{2}$ (certificates of deposit, CDs). Among them, $X_{1},X_{2},X_{3}$ are confidential and $S_{1},S_{2}$ are non-confidential. Some marginal distributions of them do not follow normal distributions. Specifically, $X_{1}$ follows normal distribution, $X_{2}$ follows exponential distribution, $X_{3}$ follows Gamma distribution, $S_{1}$ follows a normal distribution, and $S_{2}$ follows lognormal distribution. Under these circumstances, the GADP method is no longer effective. If we apply the GADP method to this dataset, the statistical information of the attributes will be affected. More details are available from Muralidhar et al. [13]. To accommodate the complexity of the original survey data, the following method was introduced, which combines the copula function with the GADP method. In probability and statistical theories, a Gaussian copula function can be used to construct the joint distribution of random variables using their marginal distributions and their Pearson’s correlation matrix to determine their dependence structure. With the use of Gaussian copulas, Sarathy et al. [21] developed the GADP method and obtained the CGADP method. Before introducing the CGADP method, we review the copula theory briefly in the following subsection.

#### Copula Function

Copula is a word from Latin that means “connection”. The definition of the copula function is as follows:

**Definition** **1.** *The function*$C:{\left[0,1\right]}^{d}\to \left[0,1\right]$*is a d-dimensional copula if*C*is a joint cumulative distribution function of a d-dimensional random vector on*${\left[0,1\right]}^{d}$*with uniform marginals*.

From Definition 1 we can create a multivariate joint distribution function for those uniformly distributed random variables using a copula function. In other words, the copula function can give a reasonable estimation of the unknown multivariate joint distribution, so it is a valuable tool for solving multivariate problems. The theoretical basis of the copula is Sklar’s theorem, which was first proposed by Sklar [22]. With the subsequent development, there are many different kinds of copula functions (e.g., Gaussian copula, t-copula, Archimedean copula, etc.) Readers can discover more copula functions from Nelsen [23]. Copula functions are widely used in various fields, especially finance. For example, Cherubini et al. [24] illustrated the theory of copulas and presented some financial applications. Below we give the definition of the Gaussian copula function, and briefly describe how to get the estimation of an unknown multivariate joint distribution by a Gaussian copula function.

**Definition** **2.** *Gaussian copula function. A p-dimensional Gaussian copula function with the correlation matrix*ρ*is defined as follows:*

(4) $$C_{p}^{Guassian}\left(u\right)=\Phi_{\rho }\left(\Phi^{-1}\left(u_{1}\right),\cdots ,\Phi^{-1}\left(u_{p}\right)\right)$$

*where*$u=\left(u_{1},\ldots ,u_{p}\right)$, $\Phi_{\rho }$*is the joint distribution function of a p-dimensional standard normal random vector whose correlation matrix is*ρ*, and*$\Phi^{-1}$*is the inverse function of the univariate standard normal distribution.*

Now, we use the Gaussian copula function as an example to illustrate how to use copulas to estimate an unknown multivariate joint distribution function. It is suggested in probability theories that all information contained in a set of random attributes is totally included in their multivariate joint distribution function. The basic idea of using a Gaussian copula function to estimate an unknown joint distribution is that the information contained in the multivariate joint distribution of random variables can be decomposed into the information contained in the marginal distribution of each attribute and the information contained in the correlation matrix between transformed attributes. Therefore, as long as the marginal distribution of each attribute and the correlation matrix between the transformed attributes are known, we can use a Gaussian copula function to obtain a multivariate joint distribution for the attributes. For example, for the five attributes $X_{1},X_{2},X_{3},S_{1},S_{2}$ in the above example, we know their marginal distributions as follows:

1. $X_{1}$ follows normal distribution, denoted by $G_{1}$;
2. $X_{2}$ follows exponential distribution, denoted by $G_{2}$;
3. $X_{3}$ follows exponential distribution, denoted by $G_{3}$;
4. $S_{1}$ follows exponential distribution, denoted by $F_{1}$; and
5. $S_{2}$ follows exponential distribution, denoted by $F_{2}$.

Their correlation matrix ρ, which is consistent with that in Muralidhar el at. [13], is shown in Table 1.

Following Muralidhar el at. [13], we choose the Gaussian copula function to create an estimation of their joint distribution. Readers can refer to Nelsen [23] to know how to choose the appropriate copula. Here, we present the estimation of the joint distribution of these five attributes using the Gaussian copula function as follows:

$$C_{p}^{Guassian}=\Phi_{\rho }\left(\Phi^{-1}\left(G_{1}\left(x_{1}\right)\right),\Phi^{-1}\left(G_{2}\left(x_{2}\right)\right),\Phi^{-1}\left(G_{3}\left(x_{3}\right)\right),\Phi^{-1}\left(F_{1}\left(s_{1}\right)\right),\Phi^{-1}\left(F_{2}\left(s_{2}\right)\right)\right)$$

In practice, if we provide the marginal distributions of all the attributes and the correlation matrix of these attributes to the statistics software R, we can generate samples from $C_{\rho }^{Gaussian}$ and perform further statistical analysis. Next, we discuss the statistical properties of the Gaussian CGADP method.

### 2.3. Gaussian Copula General Additive Data Perturbation Method

In this section, we discuss the statistical properties of Gaussian CGADP method and explain how it can be applied to real-world scenarios. Firstly, we should present an important property of all elliptical copula functions, which includes the Gaussian copula function.

**Theorem** **1.** *Let*X*be a p-dimensional random vector whose distribution can be expressed by the elliptical copula function—for example, the above Gaussian copula function*$C_{\rho }^{Guassian}$*and t-copula function*$C_{\rho ,v}^{t}$*. Let*R*be the*p×p*Spearman’s Rank correlation matrix of*X*and*ρ*be the Pearson’s correlation matrix of the*X*. Then we get:*

(5) $$\rho_{i,j}=2\sin\left(\frac{\pi r_{i,j}}{6}\right)$$

*where*$\rho_{i,j}$*is the*(i,j)*element of*ρ*, and*$r_{i,j}$*is the*(i,j)*element of*R.

According to Theorem 1, we can obtain the Pearson’s correlation matrix, which is necessary for estimating joint distribution using the copula function. Next we discuss how to use the Gaussian CGADP method step by step with an example.

#### The Gaussian CGADP Method

The first step of using the Gaussian CGADP method is to determine the marginal distribution of each attribute. As for the above example, the marginal distributions of the five attributes are normal distribution $G_{1}$, exponential distribution $G_{2}$, gamma distribution $G_{3}$, normal distribution $F_{1}$, and lognormal distribution $F_{2}$, respectively. Secondly, using these marginal distributions and the $\Phi^{-1}$, which represents the inverse of the distribution of the univariate standard normal function, we can obtain the new data $X^{*}=\left(X_{1}^{*},X_{2}^{*},X_{3}^{*}\right),S^{*}=\left(S_{1}^{*},S_{2}^{*}\right)$ by the formulas as follows:

(6) $$X_{1,i}^{*}=\Phi^{-1}\left(G_{1}\left(X_{1,i}\right)\right),\;i=1,\cdots ,N\;X_{2,i}^{*}=\Phi^{-1}\left(G_{2}\left(X_{2,i}\right)\right),\;i=1,\cdots ,N\;X_{3,i}^{*}=\Phi^{-1}\left(G_{3}\left(X_{3,i}\right)\right),\;i=1,\cdots ,N\;S_{1,i}^{*}=\Phi^{-1}\left(F_{1}\left(S_{1,i}\right)\right),\;i=1,\cdots ,N\;S_{2,i}^{*}=\Phi^{-1}\left(F_{2}\left(S_{2,i}\right)\right),\;i=1,\cdots ,N$$

The new random variables $X_{1}^{*},X_{2}^{*},X_{3}^{*},S_{1}^{*},S_{2}^{*}$ obtained by the above method have three properties: (i) all marginal distributions of them are standard normal; (ii) the joint distribution of all variables is normal; (iii) the Spearman’s Rank correlation matrix of them is consistent with that of the original five attributes, and we use the statistical software R to refer to this matrix. In other words, the new data $\left(X^{*},S^{*}\right)$ satisfies the assumptions of the GADP method. Simultaneously, based on Theorem 1, we can derive the Pearson’s correlation matrix $\rho^{*}$ of $\left(X^{*},S^{*}\right)$ based on the Spearman’s Rank correlation matrix R of $\left(X^{*},S^{*}\right)$ using the following equation:

(7) $$\rho_{i,j}^{*}=2\sin\left(\frac{\pi r_{i,j}}{6}\right)$$

where $\rho_{i,j}^{*}$ is the (i,j) element of $\rho^{*}$, and $r_{i,j}$ is the (i,j) element of R.

We apply the GADP method to the new data $\left(X^{*},S^{*}\right)$ to prevent disclosure. According to the theory and steps of the GADP method we discussed in Section 2.1, we generate the perturbed data $Y^{*}=\left(Y_{1}^{*},Y_{2}^{*},Y_{3}^{*}\right)$ from the conditional distribution $\left(X^{*}|S_{1,i}^{*},S_{2,i}^{*}\right),\;i=1,\cdots ,N$. Namely, $Y_{i}^{*}=\left(Y_{1,i}^{*},Y_{2,i}^{*},Y_{3,i}^{*}\right),\;i=1,\cdots ,N$ is a sample from the following conditional distribution:

(8) $$Y^{*}|U^{*}=U_{i}^{*}∼N\left(\mu_{X^{*}}+\Sigma_{Y^{*}U^{*}}\Sigma_{U^{*}U^{*}}^{-1}\left(U_{i}^{*}-\mu_{U^{*}}\right),\Sigma_{X^{*}X^{*}}-\Sigma_{Y^{*}U^{*}}\Sigma_{U^{*}U^{*}}^{-1}\Sigma_{U^{*}Y^{*}}\right)$$

where $U^{*}=\left(X^{*},S^{*}\right)$. As we discussed in Section 2.1, statistical information of $Y^{*}$ is consistent with that of $X^{*}$.

Finally, we transform the $Y_{i}^{*}=\left(Y_{1,i}^{*},Y_{2,i}^{*},Y_{3,i}^{*}\right)$ to $Y_{i}=\left(Y_{1,i},Y_{2,i},Y_{3,i}\right)$ using the following formulas:

(9) $$Y_{1,i}=G_{1}^{-1}\left(\Phi \left(Y_{1,i}^{*}\right)\right)\;Y_{2,i}=G_{2}^{-1}\left(\Phi \left(Y_{2,i}^{*}\right)\right)\;Y_{3,i}=G_{3}^{-1}\left(\Phi \left(Y_{3,i}^{*}\right)\right)$$

So far, we have derived the perturbed data $Y_{i}=\left(Y_{1,i},Y_{2,i},Y_{3,i}\right),\;i=1,\cdots ,N$, whose marginal distributions of the perturbed data are same as those of the original survey data X. Besides, the Pearson’s correlation matrix of the released data (Y,S) is close to that of (X,S). Following Muralidhar el at. [13], the Pearson’s correlation matrix of (Y,S) is calculated as shown in Table 2.

**Figure 2:**
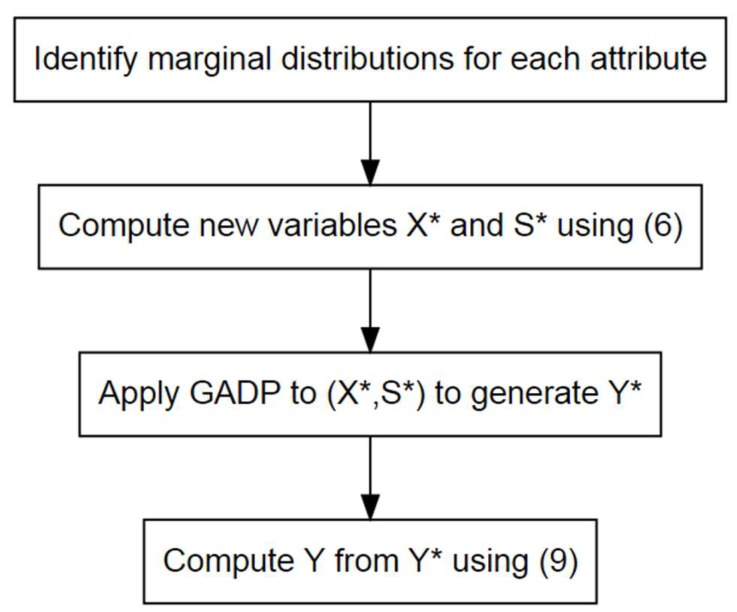
Procedure for applying CGADP.

To conclude, the procedure for applying CGADP is as shown in Figure 2.

## 3. Empirical Study Results

In this section, we illustrate the process of using the GADP and Gaussian CGADP methods by applying them to an empirical study including a set of data obtained by a questionnaire on sleep quality from 186 patients who were all Chinese woman. Some questions involved in this questionnaire are listed in Table 3. As we discussed in the previous section, both methods divide attributes into two categories (confident attributes and non-confident attributes), generate perturbed data to mask the confident attributes, then release the perturbed data and the data of non-confident attributes. For this empirical study, the answers to the first five sleep-related questions are confidential and are denoted as $X=\left(X_{1},X_{2},X_{3},X_{4},X_{5}\right)$, while the other two are non-confidential and are denoted as $S=\left(S_{1},S_{2}\right).$ To be consistent with the above, in the rest of this section we let $Y=\left(Y_{1},Y_{2},Y_{3},Y_{4},Y_{5}\right)$ denote the perturbed data and (Y,S) denote the released data. Furthermore, we compare the efficacy of the two methods.

The original survey data we obtained is shown in Table 4. Rather than every single record of the respondents, researchers are more concerned with the descriptive statistics, which are shown in Table 5 and Table 6.

To protect the privacy of all respondents, hospitals or the data owners may apply the SDC methods to mask the confident attributes of the original survey data and release the masked and non-confident data to researchers for analysis. In the following subsection, we apply GADP and Gaussian CGADP methods to the dataset.

### 3.1. Applying the GADP Method

Firstly, we need to determine the parameters of the GADP method based on Table 5 and Table 6:

(10) $$\mu_{X}=\mu_{Y}=\left(2.37,2.31,1.15,1.74,1.59\right),\mu_{S}=\left(55.44,157.69\right),$$

(11) ΣXX=ΣYY=(1    0.44611   0.17950.15901  0.10920.03790.20911 −0.05120.01160.15930.24891),

(12) ΣSS=(1 0.37191); ΣXS=ΣYS=(−0.10170.0032−0.0336−0.0569−0.09770.0592−0.02210.08070.1003−0.0900).

Under the GADP method introduced in Section 2.1, we generate the perturbed data Y according to Equations (2) and (3), as follows:

(13) $$Y|U=U_{i}∼N\left(\mu_{X}+\Sigma_{YU}\Sigma_{UU}^{-1}\left(U_{i}-\mu_{U}\right),\Sigma_{XX}-\Sigma_{YU}\Sigma_{UU}^{-1}\Sigma_{UY}\right),$$

where

(14) $$U_{i}=\left(X_{i},S_{i}\right),\;i=1,\ldots ,186.$$

Based on Equations (3) and (10), we have

(15) $$\mu_{U}=\left(\mu_{X},\mu_{S}\right)=\left(2.37,2.31,1.15,1.74,1.59,55.44,157.69\right).$$

Combining Equation (3) with Equations (11) and (12), we can get

(16) $$\Sigma_{YU}=\left(\begin{array}{ccccccc} 0.0586 & 0.0065 & 0.0540 & 0.0281 & -0.0673 & -1.9439 & 0.0389\\ 0.0065 & 0.0177 & -0.0064 & -0.0174 & -0.0138 & -0.6693 & -0.7255\\ 0.0540 & -0.0064 & 0.0587 & 0.0408 & -0.0774 & -1.4605 & 0.5661\\ 0.0281 & -0.0174 & 0.0408 & 0.0381 & -0.0578 & -0.3843 & 0.8975\\ -0.0673 & 0.0138 & -0.0774 & -0.0578 & 0.1038 & 1.6654 & -0.9551 \end{array}\right)\;\mathrm{and}$$

(17) ΣUU=(4.7629      2.21715.1867     0.66900.61852.9178    0.47320.17140.70933.9439   −0.21200.05020.51590.93723.5943  −1.9439−0.6693−1.4605−0.38431.665476.6472 0.0389−0.72550.56610.8975−0.955118.228431.3509).

We use the above information to perform GADP to obtain the perturbed data Y, and these data are released together with the non-confidential data S. Perturbed data using the GADP can be found in Table 7. By analyzing the released data (Y,S), we get the statistical information of (Y,S), which are shown in Table 8 and Table 9. 

Comparing the means and standard deviations in Table 8 with the corresponding values in Table 5, we find that there is only a small deviation in the means and standard deviations between the perturbed and original data. This is because the perturbed data are generated according to Equation (2), which helps to keep the means and standard deviations close to those of original data. However, when comparing the Pearson’s correlation matrix in Table 5 and the Spearman’s Rank correlation matrix in Table 6 with the corresponding matrix in Table 8 and Table 9, respectively, we find that many values of them become different from those of the original data. We divide these changes between the correlation matrix calculated from the original survey data and the perturbed data into two categories: (i) in which the sign of the correlation coefficient between the two attributes has changed; (ii) in which the signs of the correlation coefficients are the same, but their absolute difference is greater than a threshold. As discussed in Section 2, we expect the perturbed data would carry the same statistical information as in the original database when applying data perturbation. In this study, we set a small threshold of 0.05 to allow some variation in correlation values due to randomness in simulating samples. These two changes show that the correlation between the attributes in the original survey data are changed after using the SDC method. The first one means that if two attributes in the original survey data are positively correlated, they become negatively correlated by analyzing the perturbed data. Referring to Table 5 and Table 6, we find that there are two changes belonging to (i), which are marked with ^ in Table 8 and Table 9.

The second one means that even if the sign of the correlation coefficient between the two attributes remains unchanged, the absolute value of the correlation coefficient changes greatly. That is, the strength of the correlation between the attributes has changed. Comparing Table 6 and Table 9, we find that there are 17 changes belonging to (ii), which are marked with # if the absolute difference of the correlation is >0.05. All these biases can mislead researchers, since only the perturbed data are available to them. We see that the GADP method helps to produce perturbed data with close means and standard deviations to those of the original data, but the values in the correlation matrices differ substantially from those of the original data. The main reason for these biases is that the normal assumption of the GADP method is not satisfied in this case. The GADP method assumes normal marginals for all of the variables, which clearly is not true for this dataset with a large number of zeros observed in the survey data.

### 3.2. Applying the Gaussian CGADP m = Method

**Figure 3:**
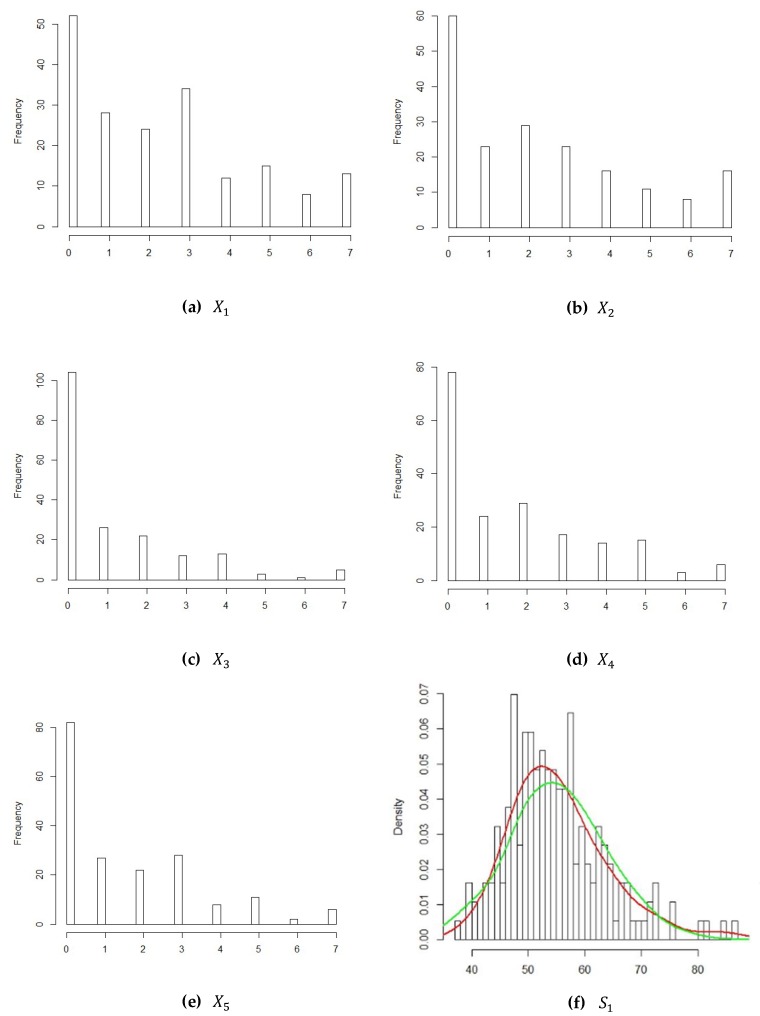

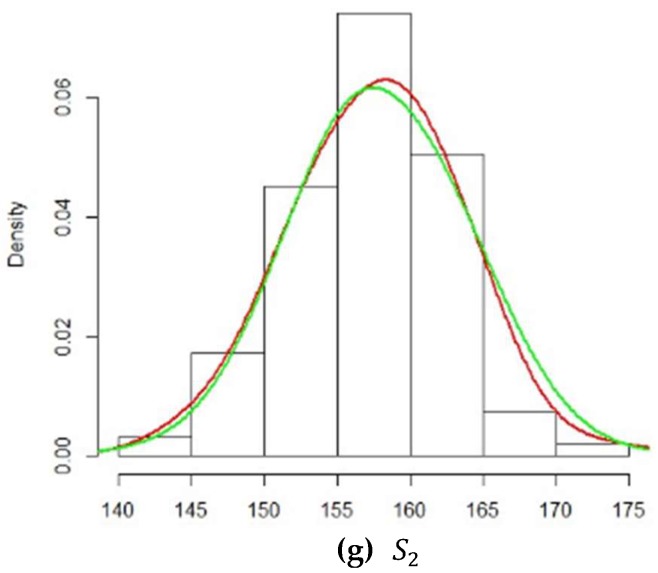
Frequency histograms of all the confidential and non-confidential data.

We should first determine the marginal distributions of attributes when we use the Gaussian CGADP method. For this empirical example, to fit the distribution of each attribute, we examine the frequency histograms using the statistical software R, and the results are shown in Figure 3.

We found that only $S_{1},S_{2}$ likely follow normal distributions. We use the Maximum-likelihood Fitting Method of Univariate Distributions, which can be implemented with R to fit the corresponding distribution for each attribute. After fitting, we use the one-sample Kolmogorov–Smirnov (K-S) test to verify the goodness of fit. The parameters, the values of K-S test and the *p*-values of K-S test, are also shown in Table 10.

**Figure 4:**
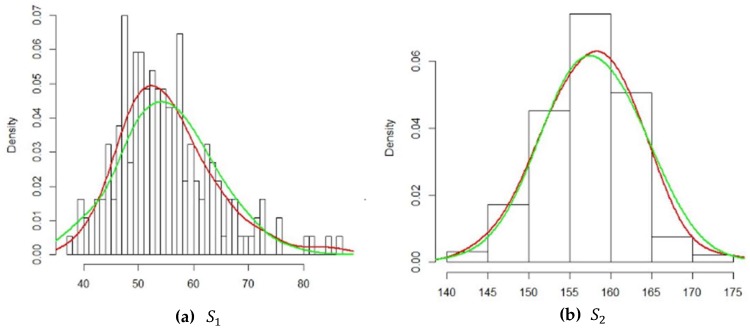
Frequency histograms of non-confidential data with a density curve.

From Table 10, we can see that all *p*-values of the Kolmogorov–Smirnov test were larger than 0.05, meaning that each of the marginal distributions should not be rejected. All parameters of each marginal distribution were estimated. In Figure 4, we compare the density curve of each attribute with the density of the corresponding fitted distribution. The red curves in (a) and (b) stand for the density of $S_{1}\;and\;S_{2}$, respectively. The green curves in (a) and (b) stand for the density of the normal distribution.

So far, we have determined the marginal distribution of $S_{1}$, $S_{2}$ as follows:

1. $S_{1}∼F_{1}=N\left(\mu_{S_{1}}=55.44,\sigma_{S_{1}}=8.75\right)$
2. $S_{2}∼F_{2}=N\left(\mu_{S_{2}}=157.69,\sigma_{S_{2}}=5.60\right)$

As for attributes $X_{1},X_{2},X_{3},X_{4},X_{5}$, it is more reasonable to assume discrete distributions. In other words, their marginal distributions cannot be normal. Now we model the confidential survey data as a discrete distribution and keep the non-confidential data as normal distribution. CGADP is based on Sklar’s Theorem, which states that the copula for the joint distribution is unique if all the marginal distributions for each variable are continuous. The mix of discrete and continuous marginals would not guarantee the uniqueness of the copula, but still we could apply CGADP by assuming that a Gaussian copula is the copula for the joint distribution in this empirical study. We first find reasonable discrete distributions to serve as the marginals for the survey data. The results of the Chi-square goodness of fit test for the survey data is shown in Table 11.

As shown in the graphics of the density plots in Figure 3, most of the values recorded in the survey data were 0, and the frequency was clearly higher than for other values. Hence, instead of simply fitting a negative binomial distribution, we fit a zero inflated negative binomial distribution (ZINB) or zero adjusted negative binomial (ZANB) as the marginals for the confidential data. The *p*-values were all higher than 0.05, indicating that the proposed distributions provide a good fit for the survey data.

We first transform the data (X,S) according to Equation (6).

(18) $$X_{1,i}^{*}=\Phi^{-1}\left(G_{1}\left(X_{1,i}\right)\right),\;i=1,\cdots ,N\;X_{2,i}^{*}=\Phi^{-1}\left(G_{2}\left(X_{2,i}\right)\right),\;i=1,\cdots ,N\;X_{3,i}^{*}=\Phi^{-1}\left(G_{3}\left(X_{3,i}\right)\right),\;i=1,\cdots ,N\;X_{4,i}^{*}=\Phi^{-1}\left(G_{4}\left(X_{4,i}\right)\right),\;i=1,\cdots ,N\;X_{5,i}^{*}=\Phi^{-1}\left(G_{5}\left(X_{5,i}\right)\right),\;i=1,\cdots ,N\;S_{1,i}^{*}=\Phi^{-1}\left(F_{1}\left(S_{1,i}\right)\right),\;i=1,\cdots ,N\;S_{2,i}^{*}=\Phi^{-1}\left(F_{2}\left(S_{2,i}\right)\right),\;\;i=1,\cdots ,N$$

According to the Definition 2, we obtain a joint distribution $C_{\rho^{*}}^{Gaussian}$ by combining all the marginal distributions, the Pearson’s correlation $\rho^{*}$ and the Gaussian copula function. The data are derived in such way have two properties: (i) the marginal distribution of each attribute is the same as the proposed marginal distributions above; (ii) the Spearman’s Rank correlation matrix of $\left(X^{*},S^{*}\right)$ is consistent with that of the original survey data (X,S). The data $U^{*}=\left(X^{*},S^{*}\right)$ meets the assumptions of the GADP method, so we apply the GADP method to $\left(X^{*},S^{*}\right)$ to derive $Y^{*}$. Then, we calculate the mean vector and the covariance matrix of $U^{*}$.

(19) $$\mu_{U^{*}}=\left(\mu_{X^{*}},\mu_{S^{*}}\right);\Sigma_{U^{*}U^{*}}=\left(\begin{array}{cc} \Sigma_{X^{*}X^{*}} & \Sigma_{X^{*}S^{*}}\\ \Sigma_{S^{*}X^{*}} & \Sigma_{S^{*}S^{*}} \end{array}\right)$$

According to assumptions of the GADP method, we have $\Sigma_{Y^{*}Y^{*}}=\Sigma_{X^{*}X^{*}},\Sigma_{Y^{*}S^{*}}=\Sigma_{X^{*}S^{*}}$. Furthermore, we have

(20) $$\left(\begin{array}{cc} \Sigma_{U^{*}U^{*}} & \Sigma_{U^{*}Y^{*}}\\ \Sigma_{Y^{*}U^{*}} & \Sigma_{Y^{*}Y^{*}} \end{array}\right)=\left(\begin{array}{ccc} \Sigma_{X^{*}X^{*}} & \Sigma_{X^{*}S^{*}} & \Sigma_{X^{*}Y^{*}}\\ \Sigma_{S^{*}X^{*}} & \Sigma_{S^{*}S^{*}} & \Sigma_{S^{*}Y^{*}}\\ \Sigma_{Y^{*}X^{*}} & \Sigma_{Y^{*}S^{*}} & \Sigma_{Y^{*}Y^{*}} \end{array}\right)$$

Based on Equation (8), we can generate $Y^{*}$ from the following distribution:

(21) $$Y^{*}|U^{*}=U_{i}^{*}∼N\left(\mu_{X^{*}}+\Sigma_{Y^{*}U^{*}}\Sigma_{U^{*}U^{*}}^{-1}\left(U_{i}^{*}-\mu_{U^{*}}\right),\Sigma_{X^{*}X^{*}}-\Sigma_{Y^{*}U^{*}}\Sigma_{U^{*}U^{*}}^{-1}\Sigma_{U^{*}Y^{*}}\right)$$

Furthermore, we obtain the perturbed data Y based on Equation (9); specifically,

(22) $$Y_{1,i}=G_{1}^{-1}\left(\Phi \left(Y_{1,i}^{*}\right)\right)\;Y_{2,i}=G_{2}^{-1}\left(\Phi \left(Y_{2,i}^{*}\right)\right)\;Y_{3,i}=G_{3}^{-1}\left(\Phi \left(Y_{3,i}^{*}\right)\right)\;Y_{4,i}=G_{4}^{-1}\left(\Phi \left(Y_{4,i}^{*}\right)\right)\;Y_{5,i}=G_{5}^{-1}\left(\Phi \left(Y_{5,i}^{*}\right)\right)$$

Finally, we combine the perturbed data Y and the data of the non-confident attributes S and release (Y,S) to researchers. Perturbed data using CGADP can be found in Table 12. Analyzing the released data (Y,S), we can derive the statistical information, which is shown in Table 13 and Table 14. Values are marked with ^ if the sign changed after perturbation, or with # if the value greatly deviated from the original data by 0.05.

Comparing means and standard deviations in Table 13 with the corresponding values in Table 5, we can easily find that the mean of the perturbed data using CGADP is slightly higher than that of the original data. The standard deviations are close to the original data, although they are not as close as those in GADP.

As for the correlation matrix in Table 13 and Table 14, we can compare values of them with Table 5 and Table 6, respectively, to find only one entry in the Pearson’s correlation matrix belonging to (i), which is marked with ^. In addition, there are 13 changes belonging to (ii), which are marked with #. This means the absolute value of these 13 correlation coefficients has been noticeably changed. We can see that there is an improvement in the closeness of the correlation matrices when using CGADP. This is because the assumed marginals are now discrete, which fits the data better than the normal marginals in GADP. The only noticeable downside of using Gaussian CGADP is that the means of the perturbed data using CGADP slightly deviate from the means of the original data. This could be due to using a mix of discrete and continuous marginals when applying CGADP.

## 4. Conclusions

Data privacy is important and the disclosure of confidential information can harm respondents and possibly the public. In addition to protecting respondents’ privacy when sensitive questions are involved during data collection [25], it is important to protect the confidentiality of data when data analysis is conducted by third parties. Statistical disclosure control (SDC) is useful for providing a quantitative balance between data utility and disclosure risk. Very few studies in public health have adopted SDC or discussed how to apply SDC in their research to protect data privacy. 

In the current research, we introduce and demonstrate the use of two useful SDC methods, a general additive perturbation method and the copula perturbation method, in an empirical study on sleeping quality among a group of patients. The results from our empirical study show that using the general additive perturbation method and the copula perturbation method help to release perturbed data of their confidential attributes and protect the privacy of patients. At the same time, these methods retain the statistical information of the original database. This allows the perturbed database to be used for further research and analysis without the need of having access to the original database and therefore the opportunities for the research and analysis of interesting but sensitive topics in public health and health care will be feasible. 

## Figures and Tables

**Table 1:** Pearson’s correlation matrix of $X_{1},X_{2},X_{3},S_{1},S_{2}$.

	$X_{1}$	$X_{2}$	$X_{3}$	$S_{1}$	$S_{2}$
$X_{1}$	1				
$X_{2}$	0.70	1			
$X_{3}$	0.80	0.75	1		
$S_{1}$	0.50	0.40	0.25	1	
$S_{2}$	0.30	0.20	0.15	0.60	1

**Table 2:** Pearson’s correlation matrix of $Y_{1},Y_{2},Y_{3},S_{1},S_{2}$.

	$Y_{1}$	$Y_{2}$	$Y_{3}$	$S_{1}$	$S_{2}$
$Y_{1}$	1				
$Y_{2}$	0.6322	1			
$Y_{3}$	0.7571	0.7216	1		
$S_{1}$	0.5025	0.3576	0.2342	1	
$S_{2}$	0.2152	0.1336	0.1000	0.4398	1

**Table 3:** Questions.

Variable	Description
$X_{1}$	Feel rested upon awakening at the end of a sleep period
$X_{2}$	Feel satisfied with the quality of your sleep
$X_{3}$	Get too much sleep
$X_{4}$	Take a nap at a scheduled time
$X_{5}$	Fall asleep at an unscheduled time
$S_{1}$	Weight
$S_{2}$	Height

Note: 0 = no days, 1 = 1 day, 2 = 2 days, 3 = 3 days, 4 = 4 days, 5 = 5 days, 6 = 6 days, 7 = every day. The respondents give answers according to his or her own situation.

**Table 4:** Original survey data.

No.	$X_{1}$	$X_{2}$	$X_{3}$	$X_{4}$	$X_{5}$	$S_{1}$	$S_{2}$
1	0	0	7	5	7	56	165
2	0	0	0	0	0	48	152
⋮	⋮	⋮	⋮	⋮	⋮	⋮	⋮
⋮	⋮	⋮	⋮	⋮	⋮	⋮	⋮
185	1	3	1	0	0	72	162
186	7	1	0	1	1	47	149

**Table 5:** Statistical information from the original survey database.

Summary Statistics			Pearson’s Correlation Matrix						
Mean	Std		$X_{1}$	$X_{2}$	$X_{3}$	$X_{4}$	$X_{5}$	$S_{1}$	$S_{2}$
2.37	2.18	$X_{1}$	1						
2.31	2.28	$X_{2}$	0.4461	1					
1.15	1.71	$X_{3}$	0.1795	0.1590	1				
1.74	1.99	$X_{4}$	0.1092	0.0379	0.2091	1			
1.59	1.90	$X_{5}$	−0.0512	0.0116	0.1593	0.2489	1		
55.44	8.75	$S_{1}$	−0.1017	−0.0336	−0.0977	−0.0221	0.1003	1	
157.69	5.60	$S_{2}$	0.0032	−0.0569	0.0592	0.0807	−0.0900	0.3719	1

**Table 6:** Spearman’s Rank correlation matrix of the original survey database.

	$X_{1}$	$X_{2}$	$X_{3}$	$X_{4}$	$X_{5}$	$S_{1}$	$S_{2}$
$X_{1}$	1						
$X_{2}$	0.4680	1					
$X_{3}$	0.2017	0.2254	1				
$X_{4}$	0.1285	0.0787	0.1985	1			
$X_{5}$	−0.0300	0.0621	0.1690	0.3009	1		
$S_{1}$	−0.0863	−0.0437	−0.0698	−0.0355	0.1090	1	
$S_{2}$	0.0039	−0.0531	0.0123	0.0954	−0.0470	0.3482	1

**Table 7:** Perturbed data using the GADP method.

No.	$Y_{1}$	$Y_{2}$	$Y_{3}$	$Y_{4}$	$Y_{5}$	$S_{1}$	$S_{2}$
1	4.6587	2.7093	−2.0323	3.6562	5.9077	56	165
2	4.4505	5.0854	0.7921	−1.7925	−2.3083	48	152
⋮	⋮	⋮	⋮	⋮	⋮	⋮	⋮
⋮	⋮	⋮	⋮	⋮	⋮	⋮	⋮
185	2.0445	2.0600	1.2364	0.4676	2.6754	72	162
186	4.3816	4.2980	2.8277	2.9120	2.6487	47	149

**Table 8:** Statistical information of the perturbed data using the GADP method.

Summary Statistics			Pearson’s Correlation Matrix						
Mean	Std		$Y_{1}$	$Y_{2}$	$Y_{3}$	$Y_{4}$	$Y_{5}$	$S_{1}$	$S_{2}$
2.43	2.14	$Y_{1}$	1						
2.43	2.39	$Y_{2}$	0.4994 ^#^	1					
0.93	1.72	$Y_{3}$	0.1741	0.2190 ^#^	1				
1.61	2.03	$Y_{4}$	0.1834 ^#^	0.1141 ^#^	0.1503 ^#^	1			
1.58	1.88	$Y_{5}$	0.0315 ^^^	0.0548	0.2573 ^#^	0.3349 ^#^	1		
55.44	8.75	$S_{1}$	−0.1022	−0.1272 ^#^	−0.1063	−0.0614	0.1642 ^#^	1	
157.69	5.60	$S_{2}$	0.0375	−0.1244 ^#^	0.0808	0.0759	−0.0722	0.3719	1

Note: values are marked with ^ if the sign changed after perturbation, or with # if the absolute difference of the value is >0.05.

**Table 9:** Spearman’s Rank correlation matrix of the perturbed data using the GADP method.

	$Y_{1}$	$Y_{2}$	$Y_{3}$	$Y_{4}$	$Y_{5}$	$S_{1}$	$S_{2}$
$Y_{1}$	1						
$Y_{2}$	0.4892	1					
$Y_{3}$	0.1790	0.2300	1				
$Y_{4}$	0.2117 ^#^	0.1306 ^#^	0.1873	1			
$Y_{5}$	0.0109 ^^^	0.0436	0.2704 ^#^	0.3062	1		
$S_{1}$	−0.0994	−0.1151 ^#^	−0.1405 ^#^	−0.0163	0.2163 ^#^	1	
$S_{2}$	0.0488	−0.0800	0.1086 ^#^	0.0892	−0.0788	0.3482	1

Note: values are marked with ^ if the sign changed after perturbation, or with # if the absolute difference of the value is >0.05.

**Table 10:** Fitting distribution of $S_{1},S_{2}$ and goodness of fit test.

	Distribution	Parameters		Test Value	*p*-Value
$S_{1}$	Normal	μ = 55.44	σ = 8.73	0.0995	0.0502
$S_{2}$	Normal	μ = 157.69	σ = 5.59	0.0605	0.5029

**Table 11:** Fitting distribution of $X_{1},X_{2},X_{3},X_{4},X_{5}$ and goodness of fit test.

	Distribution	Parameters			Test Value	*p*-Value
$X_{1}$	ZANB	μ = 3.0331	σ = 0.1244	π = 0.2796	9.04	0.2498
$X_{2}$	ZINB	μ = 3.1563	σ = 0.1388	π = 0.2692	5.2487	0.6296
$X_{3}$	ZINB	μ = 2.1153	σ = 0.2780	π = 0.4561	6.2476	0.5112
$X_{4}$	ZANB	μ = 2.7233	σ = 0.1095	π = 0.4194	6.3010	0.5051
$X_{5}$	ZINB	μ = 2.5237	σ = 0.1322	π = 0.3694	9.2857	0.2328

Note: π is the parameter for a probability of zero in a zero inflated/adjusted model.

**Table 12:** Perturbed data using the CGADP method.

No.	$Y_{1}$	$Y_{2}$	$Y_{3}$	$Y_{4}$	$Y_{5}$	$S_{1}$	$S_{2}$
1	4	5	0	2	4	56	165
2	3	1	2	0	2	48	152
⋮	⋮	⋮	⋮	⋮	⋮	⋮	⋮
⋮	⋮	⋮	⋮	⋮	⋮	⋮	⋮
185	1	0	2	0	0	72	162
186	0	0	0	3	6	47	149

**Table 13:** Statistical information of the perturbed data using the CGADP method.

Summary Statistics			Pearson’s Correlation Matrix						
Mean	Std		$Y_{1}$	$Y_{2}$	$Y_{3}$	$Y_{4}$	$Y_{5}$	$S_{1}$	$S_{2}$
2.90	2.02	$Y_{1}$	1						
2.98	2.14	$Y_{2}$	0.4975 ^#^	1					
1.84	1.61	$Y_{3}$	0.1793	0.2930 ^#^	1				
2.42	1.85	$Y_{4}$	0.2327 ^#^	0.1283 ^#^	0.3395 ^#^	1			
2.01	1.80	$Y_{5}$	−0.0543	0.0294	0.1530	0.3484 ^#^	1		
55.44	8.75	$S_{1}$	−0.1179	−0.1061 ^#^	−0.1382	0.0436 ^^^	0.0744	1	
157.69	5.60	$S_{2}$	0.0418	−0.0871	0.0140	0.1057	−0.0500	0.3719	1

Note: values are marked with ^ if the sign changed after perturbation, or with # if the absolute difference of the value is >0.05.

**Table 14:** Spearman’s Rank correlation matrix of the perturbed data using the CGADP method.

	$Y_{1}$	$Y_{2}$	$Y_{3}$	$Y_{4}$	$Y_{5}$	$S_{1}$	$S_{2}$
$Y_{1}$	1						
$Y_{2}$	0.5122	1					
$Y_{3}$	0.1969	0.2902 ^#^	1				
$Y_{4}$	0.2359 ^#^	0.1636 ^#^	0.3282 ^#^	1			
$Y_{5}$	−0.0195	0.0588	0.1953	0.3304	1		
$S_{1}$	−0.1211	−0.0873	−0.1357 ^#^	−0.0108	0.0306 ^#^	1	
$S_{2}$	0.0525	−0.0765	0.0264	0.1168	−0.0300	0.3482	1

Note: values are marked with ^ if the sign changed after perturbation, or with # if the absolute difference of the value is >0.05.
